# Homolog of protein kinase Mζ maintains context aversive memory and underlying long-term facilitation in terrestrial snail *Helix*

**DOI:** 10.3389/fncel.2015.00222

**Published:** 2015-06-22

**Authors:** Pavel M. Balaban, Matvey Roshchin, Alia Kh. Timoshenko, Alena B. Zuzina, Maria Lemak, Victor N. Ierusalimsky, Nikolay A. Aseyev, Aleksey Y. Malyshev

**Affiliations:** ^1^Institute of Higher Nervous Activity and Neurophysiology of the Russian Academy of SciencesMoscow, Russia; ^2^Biology Department, Lomonosov Moscow State UniversityMoscow, Russia

**Keywords:** invertebrates, reconsolidation, memory blockade, withdrawal behavior, memory maintenance, contextual fear conditioning

## Abstract

It has been shown that a variety of long-term memories in different regions of the brain and in different species are quickly erased by local inhibition of protein kinase Mζ (PKMζ), a persistently active protein kinase. Using antibodies to mammalian PKMζ, we describe in the present study the localization of immunoreactive molecules in the nervous system of the terrestrial snail *Helix lucorum*. Presence of a homolog of PKMζ was confirmed with transcriptomics. We have demonstrated in behavioral experiments that contextual fear memory disappeared under a blockade of PKMζ with a selective peptide blocker of PKMζ zeta inhibitory peptide (ZIP), but not with scrambled ZIP. If ZIP was combined with a “reminder” (20 min in noxious context), no impairment of the long-term contextual memory was observed. In electrophysiological experiments we investigated whether PKMζ takes part in the maintenance of long-term facilitation (LTF) in the neural circuit mediating tentacle withdrawal. LTF of excitatory synaptic inputs to premotor interneurons was induced by high-frequency nerve stimulation combined with serotonin bath applications and lasted at least 4 h. We found that bath application of 2 × 10^−6^ M ZIP at the 90th min after the tetanization reduced the EPSP amplitude to the non-tetanized EPSP values. Applications of the scrambled ZIP peptide at a similar time and concentration didn’t affect the EPSP amplitudes. In order to test whether effects of ZIP are specific to the synapses, we performed experiments with LTF of somatic membrane responses to local glutamate applications. It was shown earlier that serotonin application in such an “artificial synapse” condition elicits LTF of responses to glutamate. It was found that ZIP had no effect on LTF in these conditions, which may be explained by the very low concentration of PKMζ molecules in somata of these identified neurons, as evidenced by immunochemistry. Obtained results suggest that the *Helix* homolog of PKMζ might be involved in post-induction maintenance of long-term changes in the nervous system of the terrestrial snail.

## Introduction

It has been shown that a constitutively active fragment of the mammalian atypical protein kinase Cζ protein kinase Mζ (PKMζ) plays a critical role in the persistence of long-term potentiation (LTP) in the mammalian hippocampus (Ling et al., 2002; Pastalkova et al., 2006), as well as in several forms of mammalian memory (Serrano et al., 2005; Shema et al., 2007; Gámiz and Gallo, 2011; recently reviewed in Glanzman, 2013). The ability of PKMζ to maintain LTP and memory is due to its unique structure as an autonomously active protein kinase C isoform (Sacktor et al., 1993; Sacktor, 2011). PKMζ protein persistently increases in the CA1 area of the hippocampus during LTP, and this increase correlates with the extent and duration of synaptic potentiation during LTP maintenance (Osten et al., 1996). The persistent activity of PKMζ is both necessary and sufficient for maintaining synaptic LTP (Ling et al., 2002) and long-term memory storage (Pastalkova et al., 2006; Shema et al., 2011). Recently shown localization of PKMζ within postsynaptic densities and spines (Hernández et al., 2013) supports the previously proposed (Sajikumar et al., 2005; Sacktor, 2011) “synaptic autotagging” model for explaining how this kinase stores and maintains the long-term memory.

Recent findings in invertebrate model organisms show an evolutionarily conserved role of atypical PKCs, homologous to mammalian PKMζ, in memory acquisition and maintenance (Drier et al., 2002; Cai et al., 2011). An atypical PKC has been cloned from the nervous system of *Aplysia* (Bougie et al., 2009). This *Aplysia* PKC, PKC Apl III, can undergo proteolytic cleavage by calpain, thereby yielding a PKM fragment, PKM Apl III. It is also shown that 5-HT appears to activate PKM Apl III in motor neurons of *Aplysia* (Villareal et al., 2009). Recently it was demonstrated that long-term memory in *Aplysia* is maintained via a positive-feedback loop involving PKM Apl III-dependent protein phosphorylation (Cai et al., 2011).

In the present study, using as an animal model, a phylogenetically advanced pulmonate terrestrial snail *Helix*, which is capable of demonstrating associative forms of learning, and has well described neural circuits underlying its behavior (Balaban, 2002), we have investigated: (a) whether a homolog of PKMζ exists in the neural transcriptome of this animal; (b) where this molecule is located in the nervous system; (c) whether the inhibitor of PKMζ blocks the long-term (days) associative aversive memory in behavioral experiments; and (d) whether the inhibitor of PKMζ blocks the long-term synaptic changes (hours) in identified premotor interneurons triggering the withdrawal responses.

## Methods

Experiments were carried out in adult snails *Helix lucorum L*. (Crimea population) weighing 20–30 g. All animals were kept in terraria at temperature 22 ± 2°C, in a 12:12 light/dark cycle. The snails were kept in an active state at least 2 weeks before the experiment in a wet environment and were fed regularly with cabbage. Two days before the training session, the snails were deprived of food. Each snail was used in only one series of experiments. In total scores of 43, animals that survived the training and testing procedures and were in good health at least a week after the end of experiment were used for statistical evaluation in behavioral experiments. Isolated central nervous system (CNS) was used for electrophysiological experiments. Details of preparation and identification of neurons, are given elsewhere (Ierusalimsky et al., 1992; Balaban, 2002; Malyshev and Balaban, 2002). Injection of cold isotonic MgCl_2_ was made before the CNS isolation to minimize pain. Experimental procedures were in compliance with the Guide for the Care and Use of Laboratory Animals published by the National Institutes of Health, and the protocol was approved by the Ethical Committee of the Institute of Higher Nervous Activity and Neurophysiology of Russian Academy of Sciences.

### Behavioral Experiments

#### Apparatus and Analysis of Behavior

In the experimental set-up (Context 1), the snail was tethered by its shell in a manner allowing it to crawl on a ball that rotated freely in a water solution containing 0.01% NaCl (Figure 3A, right inset). The ball was covered with aluminium foil to complete an electrical circuit between the animal’s foot and a carbon electrode placed in the water. Electric shock was delivered using a 1–4 mA, 1 s current through a macroelectrode applied manually to the dorsal surface of the snail’s foot (Figure 3A). Punctate mechanical stimuli were applied with calibrated von Frey hairs, permitting delivery of pressures ranging from 6 (estimated as weak) to 68 gr/mm^2^ (estimated as noxious). After several pilot series, the behavioral response, the intensity (25 gr/mm^2^), and the location of tactile stimulation were chosen. Ommathophores (posterior tentacles) withdrawal in response to chosen intensity of tactile stimulation of the rostral part of skin 4–5 mm behind the posterior tentacles was at the level of 10–30% of maximal in normal animals. In pilot experiments, it was shown that responses to such test stimulation were sensitized after noxious stimuli, and this part of the foot skin was chosen as the standard place for tactile stimulation. An investigator, blind to the experimental histories of the animals, applied the tactile stimuli to the snail’s skin and video recorded the tentacle withdrawal. To quantify and average the results, we analyzed off-line the distance between tip and base of the tentacle and scored the withdrawal amplitude as a percentage of initial length of the tentacle in each trial.

### Learning and Reminder Protocol

Before training, each snail was exposed for 30 min daily for 2 days to the experimental set-up. Then the first test session (T) was performed for all groups (first day, Figure 3A). Blind testing was performed for each snail in two alternating contexts (Context 1 was a ball floating in water, while the Context 2 was a flat glass similar to glass of terrarium where the snails were kept between the experimental sessions, see inset on Figure 3A). After obtaining the pre-training scores, all groups of snails received five electrical shocks per day with 20–30 min intervals for 10 days in Context 1 alone. Current magnitude was individually chosen for each snail so that a complete withdrawal of the anterior part of the body was observed in response to a shock. No testing was performed during the training session. On the second day after completion of the training session (animals were fed during the rest period in terrarium), the responsiveness to the same test tactile stimuli (T1, Figure 3A) was compared in all parallel groups of snails. The order in which the animals were tested in each context was randomized.

Next day after the second test session (T1), one group of snails (G2) was reminded of training by placing the snails for 20 min (Reminder) in the same Context 1 where they were previously shocked (on the ball, Figure 3A). Twenty minutes before the reminder, the snails were injected either with ZIP or scrambled ZIP (scrZIP, 0.4 mg in 0.2 ml of saline plus 0.5 ml of saline to equalize volume per snail weighing 20–30 g). On the second day after a session of drug injections or “reminding”, the third test session (T2) was performed for all parallel groups in two different contexts (detailed protocol in Balaban et al., 2011, 2014).

#### Drugs and Injections

ZIP (TOCRIS) and scrambled ZIP were dissolved in sterile Ringer saline [in mM: 100 NaCl, 4 KCl, 7 CaCl_2_, 5 MgCl_2_, and 10 Tris-HCl buffer (pH 7.8)]. Estimated final concentrations in the hemolymph of free behaving animals of ZIP and scrZIP were 2 × 10^−6^M. Selected concentrations were effective in our electrophysiological experiments in snails without obvious toxic effects. For calculating final concentrations in the nervous system, each gram of the snail body weight was scored as 1 ml.

Drugs for *in vivo* injections were prepared in deionized water as a stock solution at a concentration 28.6-fold greater than required. Because the snails used in these experiments were comparable in weight (20g ± 2), 0.7 ml of the drug solutions were injected into the hemocoel, thereby achieving a required concentration in the animals’ body (0.7 ml × 28.*6* = 20 ml).

Intracoelomic injections were performed with a fine needle via an insensitive part of the foot skin normally hidden under the shell. During injections, the snails stopped locomotion and lowered the ommatophores, which was most likely due to the experimenter raising the shell. However, the snails never showed a generalized withdrawal into the shell.

### Electrophysiological Experiments

Intracellular recordings from isolated brain ganglia were made using standard electrophysiological techniques. Identified withdrawal premotor interneurons of the parietal ganglia (Pa3 and Pa2; Balaban, 2002) were penetrated with sharp glass microelectrodes filled with 2 M potassium acetate (tip resistance, 15–20 MOhm). The cutaneal and intestinal nerves were stimulated via plastic suction electrodes with 3 ms stimuli. Intensity of stimuli was adjusted in each experiment to elicit complex EPSPs of 5–8 mV amplitude. Intracellular signals were recorded with preamplifiers (Axoclamp 2B, Axon Instruments, CA, USA), digitized, and stored on a computer (Digidata 1400A A/D converter and Axoscope 10.0 software, both from Axon Instruments, CA, USA).

In the first series of experiments (homosynaptic facilitation) we used test stimulation of the cutaneal nerve, and potentiation was elicited by tetanization of the same cutaneal nerve associated with bath serotonin application. Experimental protocol started with five test stimuli with a 10 min interstimulus interval, followed by tetanization (three high-frequency 10 Hz trains of stimuli; duration of each train 1 min; with 5 min intervals between trains; test stimulus amplitude was increased 10 fold), followed by posttetanic testing with single stimuli of normal amplitude and a 10 min interstimulus interval for several hours. Serotonin was applied to the experimental bath just before each tetanization train to a final bath concentration of 10^−5^ M with washout in 4 min after each tetanization train. Ninty minutes after the last tetanization train, the saline flow (0.1 ml/min) of the perfusion system was switched off, and ZIP or scrZIP stock solution was applied to a final bath concentration of 2 × 10^−6^ M.

In the second series of experiments (heterosynaptic facilitation) we also used test stimulation of the second cutaneal nerve but potentiation was elicited by tetanization of the intestinal nerve associated with serotonin application to the experimental bath. Otherwise, experimental protocol was identical to the described protocol above for homosynaptic facilitation.

In the third series (artificial synapse) we used two intracellular electrodes, one for recording and another for stimulation. Before recording, somata of the same premotor parietal giant interneurons were synaptically isolated by cutting the ganglia neuropile at a distance of 400 micrometers from the soma where the single giant neurite of these cells starts branching (our unpublished observations). All synaptic inputs of parietal withdrawal interneurons are contained in the neuropil of parietal ganglia (Balaban, 2002), and were cut out in this type of preparation, therefore diminishing possible polysynaptic effects of transmitter applications. Input resistance of the cell was monitored using fixed amplitude hyperpolarization current impulses of 2 s duration. In this series glutamate (Glu) was dissolved to a concentration of 2 × 10^−3^ M in Ringer solution with 0.15% of vital dye Fast Green and sucked in the glass pipette with ~1 μm tip. Aliquots were stored in a freezer and were warmed at room temperature for at least 30 min before use. Applications of Glu to the neural membrane were made using picoinjector (PV830 PicoPump, WPI) and pressure 70–80 psi, adjusting minimal pulse duration in order to get stable responses in a neuron with 3–10 mV amplitude. Preparations in this series of experiments were perfused (2 ml/min, bath volume 1.5 ml) using a 1 mm suction tube located close to the recorded cell and pipette with Glu, thus washing out the Glu in less than 0.5 s after the application. This timing was estimated visually with a Fast Green applied together with Glu. After the first 15 applications of Glu with 1/min intervals, serotonin was added three times in final bath concentration 10^−5^ M for 10 min, then 10 min washout. During all this time the Glu was applied repeatedly. ZIP was added 90 min after the end of the last serotonin application. After ZIP application, the perfusion system was switched to the closed cycle.

### Transcriptome Analysis

Transcriptome of *Helix lucorum taurica L*. was prepared from eight nervous systems processed separately on Illumina HiSec 2000. We extracted RNA from each sample, using an RNAaquous™ micro kit (Ambion, Austin, TX, USA). The quality and quantity of RNA were assessed using an Agilent 2100 Bioanalyzer (Agilent Technologies, CA, USA). RNA samples were subsequently used in cDNA library construction and Illumina sequencing. DNA was processed as described in the TruSeq DNA Sample Preparation Guide (Illumina). The library was quantified using fluorimetry with Qubit (Invitrogen) and real-time PCR and diluted up to final concentration of 8 pM. The diluted library was clustered on a paired-end flowcell (TruSeq PE Cluster Kit v3) using a cBot instrument and sequenced using a HiSeq2000 sequencer with the TruSeq SBS Kit v3-HS with a read length of 101 bp from each end. Paired-end libraries with 250-bp inserts were constructed with read length 100 + 100 bp and ~200 mln reads in total. The transcriptome assembly was constructed *de novo* using Trinity software (Grabherr et al., 2011). Multiple sequence alignment (MSA) was constructed by DIALIGN-PFAM online tool with default parameters (Al Ait et al., 2013).

### Immunohistochemistry

**Figure 1:**
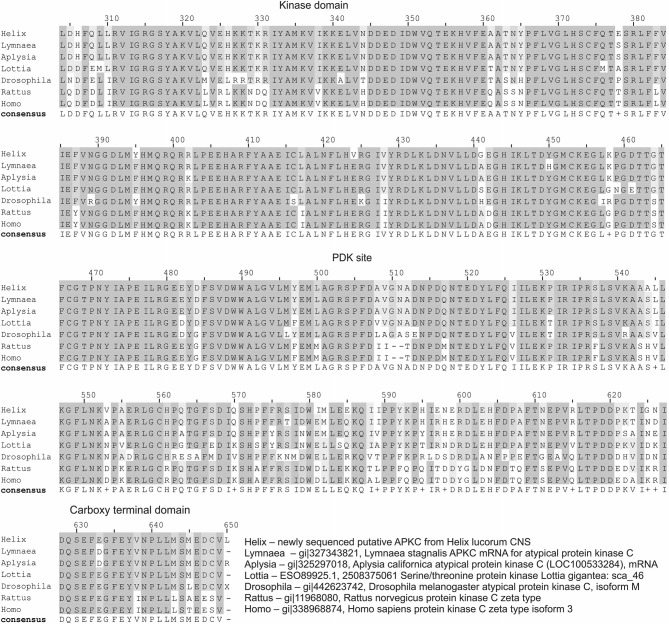
**Homology of *Helix* atypical PKC to PKMζ of different animals**. Multiple sequence alignment (MSA) of newly partially sequenced putative atypical PKC (will be available from GenBank under accession number KM875662) from *Helix lucorum* central nervous system (CNS) with putative homologs. This MSA was constructed by DIALIGN-PFAM online tool with default parameters (Al Ait et al., 2013). Amino acids conserved in aligned sequences are shaded. The domains are indicated by names above the regions. All sequences from Genbank, accession numbers are provided at the right corner of figure. For this alignment all nucleotide sequences were translated *in silico* with proper ORF. *Helix* sequence shows high homology with *Lymnaea* (94% aa identity) and *Aplysia* (91% aa identity) sequences (BLASTP). PDK site—phosphoinositide-dependent kinase site; *Helix*—newly sequenced putative a PKC from *Helix lucorum* CNS; Lymnaea—gi|327343821, *Lymnaea stagnalis* a PKC mRNA for atypical protein kinase C; Aplysia—gi|325297018, *Aplysia californica* atypical protein kinase C (LOC100533284), mRNA; Lottia—ESO89925.1, 2508375061 Serine/threonine protein kinase Lottia gigantea: sca_46; Drosophila—gi|442623742, *Drosophila melanogaster* atypical protein kinase C, isoform M; Rattus—gi|11968080, *Rattus norvegicus* protein kinase C zeta type; Homo—gi|338968874, *Homo sapiens* protein kinase C zeta type isoform 3.

Two commercially available antibodies to PKMζ were selected: sc-216, and sc-11781 (Santa Cruz Biotechnology). Both were produced to the highly conservative (C-terminus) part of PKMζ (see Figure 1). The brains of eight adult snails were subjected to the immunochemical procedure, as well as the individually identified four withdrawal interneurons mechanically isolated with the primary neurite. The brains were fixed in 4% paraformaldehyde in 0.1 PBS. The duration of fixation was 2 h at 4°C and it was followed by washes in PBS. The brains were mounted in Paraplast and sectioned at a thickness of 10 μm. Prior to incubation with the primary antibody solution, sections were washed in the blocking solution for 2 h. The blocking solution contained 0.5% Triton X-100, 0.01% sodium azide, 5% normal goat serum (Sigma) and 1% BSA (Sigma) in PBS. The staining procedure was: primary antibody for 24–48 h; wash for 2 h; secondary antibody for 24 h; wash for 2 h. The secondary antibody was either Alexa-488 goat anti-rabbit (sc-216) or Alexa-546 goat anti-mouse (sc-17781) conjugated antibody. Finally, the sections were embedded in Aqua Poly/Mount (Polyscience). Cultured neurons were processed as a whole-mount. The preparations were examined with an AxioPlan microscope (Zeiss, Germany) supplied with the program KS-300 for analysis of visual images. To test the specificity of immunostaining, the primary antiserum was omitted. No staining resulted in this series.

### Statistical Evaluation of Data

Blind testing was performed at different time intervals as shown in inset of Figure 3. Comparison between groups was made only for parallel groups of animals in one experimental series. We used nonparametric Mann-Whitney rank sum test to compare performance of two groups of snails, and Wilcoxon signed-rank test was used for comparison of performance of the same group. On all figures significant differences in performance are indicated.

## Results

### *Helix* Sequence Homology to the *Aplysia* and Mammalian PKMζ

Transcriptome of *Helix lucorum taurica* L. was prepared from eight nervous systems processed separately on Illumina HiSec 2000. Paired-end libraries with 250-bp inserts were constructed with read length 100 + 100 bp and ~200 mln reads in total. The transcriptome assembly was constructed *de novo* using Trinity software (Grabherr et al., 2011). The search resulted in finding many described genes of mollusks, and among them a homolog of the *Aplysia* atypical PKC with high identity in amino acids sequence (Figure 1). *Helix* atypical PKC sequence showed high homology with pulmonate *Lymnaea* (94% aa identity) and opistobranch *Aplysia* (91% aa identity) molluskan sequences.

### Localization of PKMζ in the Nervous System

**Figure 2:**
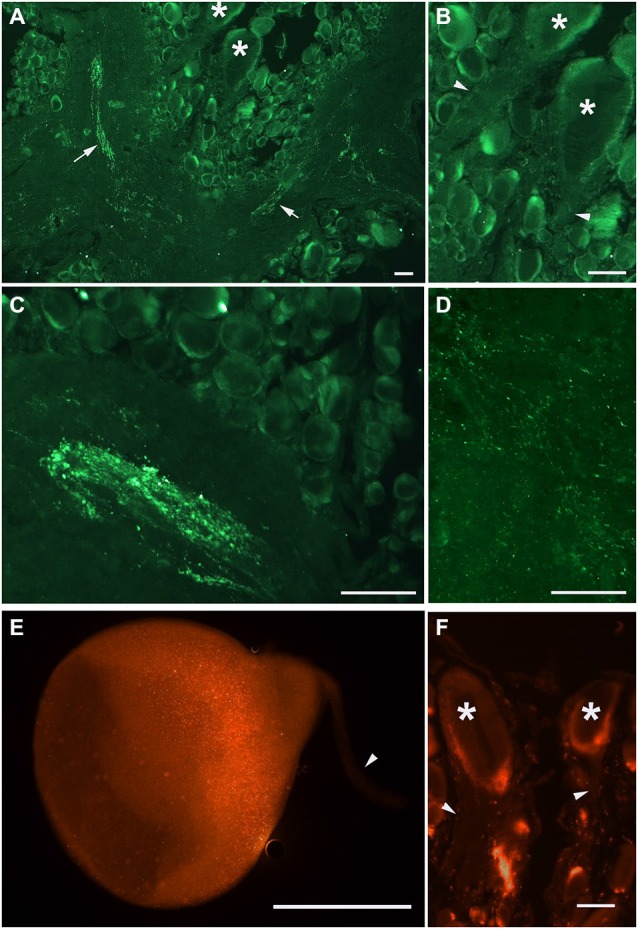
**PKMζ immunoreactivity pattern in the 10 μm sections of *Helix* brain**. Distribution of PKMζ in the nervous system of *Helix* was revealed with commercially available antibodies to highly conservative PKMζ sites. **(A–D)** Staining with sc-216 antibody, **E–F**: staining with sc-17781 antibody. **(A)** Parietal ganglia. Arrows point to the immunoreactive tracts, asterisks on **(A,B)** and **(F)** mark the cell bodies of giant parietal interneurons for the withdrawal behavior. **(B)** Same section at higher amplification. Arrowheads point to the interneurons’ primary neurites (immunoreactivity is absent). **(C)** Immunoreactive elements in the neuropil of pleural ganglia. **(D)** Immunoreactive varicosities in the neuropil of parietal ganglia. **(E)** Cultured giant parietal interneuron. **(F)** Section of the parietal ganglia, asterisks mark the cell bodies of giant parietal interneurons. In **(E)**, and **(F)** arrowheads point to the primary neurites. Scale bar 100 μm.

Our next step was aimed to analyze the distribution of PKMζ in the nervous system of *Helix*. We selected two commercially available antibodies (sc-216, and sc-11781) both produced to the highly conservative (C-terminus) part of PKMζ (see Figure 1), and immunochemically analyzed distribution of the PKMζ-reactive sites. Mainly, the results were similar with both antibodies used. It appeared that a small amount of immunoreactive fluorescence can be seen in cytoplasm of most neurons including the parietal giant interneurons used in electrophysiological experiments (stars on Figures 2A,B,F). No fluorescence was observed in large neurites of these cells (arrowheads on Figures 2B,F), while in the neuropil regions glomeruli-like areas with intensive specific fluorescent small blobs and neurite-like conglomerates were observed (Figures 2C,D). Obtained results suggest that the PKMζ-immunoreactive molecules are present in neurons of *H. lucorum*, both in cell bodies and in places where the majority of synapses are supposed to be present. In cultured giant parietal neurons, no immunoreactivity was observed in the primary neurites (arrowhead on Figure 2E), while the immunoreactive product in the cell body was detected in small granules (Figure 2E).

### Behavioral Experiments

**Figure 3:**
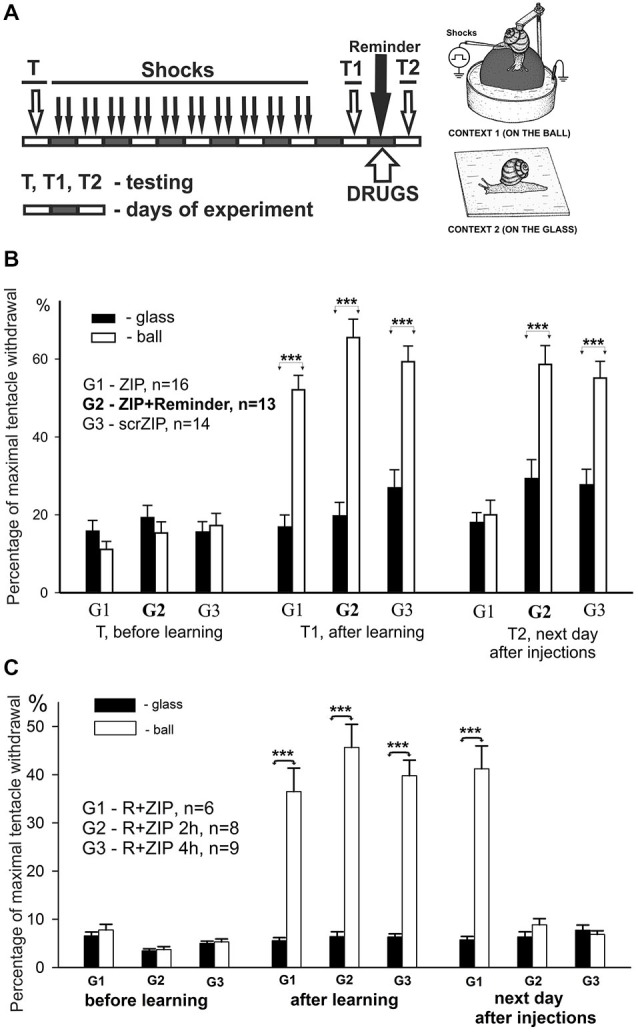
**ZIP injection impairs long-term aversive context memory in freely behaving animals. (A)** Protocol of training resulting in long-term associative memory about context in which animals were shocked, inset on the right—two contexts, “ball” and “glass”. Each block represents a day of experimental session. Snails received electric shocks only on the ball, testing always was performed in both contexts before (T), on the second day after 8 days of shocks (T1), and on the next day after reminding and injections (T2). **(B)** Averaged amplitudes (±SEM) of withdrawal responses in three groups of snails measured in two different contexts: on the ball (reinforced context) and on the glass. Group1 (G1), *n* = 16; Group2 (G2), *n* = 13; Group3 (G3), *n* = 14. Group 1 was injected with ZIP without the reminder, Group 2 with ZIP 20 min before the reminder, Group 3 with scrZIP without the reminder. Y axis—normalized amplitude of tentacle withdrawal in % of the length before the test. Significance of differences in response amplitudes in two contexts was estimated for each group using Wilcoxon Signed Rank Test. ***p < 0.001. Results showed high significance of differences in two contexts after learning (T1), complete disappearance of context memory in G1 injected with ZIP, and maintenance of memory in G2 and G3. Results suggest absence of nonspecific ZIP effects, because G2 and G3 demonstrated excellent memory, but evidence to a necessity of uninhibited PKMz for maintenance of context memory. **(C)** Averaged amplitudes (±SEM) of withdrawal responses in three groups of snails scored in two different contexts. In this behavioral experiment 3 groups of snails (G1, G2, G3) were trained and tested similarly to experiment on **(B)**, but all 3 groups received after T1 a Reminder+ZIP injections with different timing: 20 min before the Reminder, 2 h after the Reminder (G2), 4 h after the Reminder (G3). Testing on the next day after Reminder+injections showed excellent maintenance of memory in G1 (similar to obtained in G2 in experiment shown on “**B**”), and disappearance of significant context memory in G2 injected with ZIP 2 h after the Reminder, and complete disappearance of memory in G3 injected with ZIP 4 h after the Reminder. Results suggest that ZIP can be effective in conditions of reconsolidation when a new memories/PKMz molecules are supposed to be formed if the timing of ZIP effect is compatible with timing of new molecules of PKMz (2–4 h after reconsolidation procedure).

In this series of experiments, we have investigated involvement of PKMζ in maintenance of contextual memory in terrestrial snail *Helix*. Three groups of snails were randomly tested in two different contexts (ball and glass, see inset on Figure 2A) before the training session (T, see protocol in Figure 3A). Percentage of maximal withdrawal to tactile stimulation was scored. Then the snails were trained (shocked) for 10 days to remember the context in which they were shocked (Context 1—on the ball) and tested with 1 day rest interval for aversive context memory (T1 on Figure 3A). After that, group 1 was injected with ZIP without a reminder, group 2 was injected with ZIP 30 min before the reminder, and was reminded (20 min in noxious context, no shocks during reminder) of the context in which they were shocked. Group 3 was injected with scrZIP without the reminder. All groups were tested 24 h later for maintenance of context memory (T2, Figure 3A).

Prior to the training session, the behavioral responses in two contexts did not differ significantly in all groups (panel “before learning” in Figure 3B). On the second day after a 10-day session of electric shocks in Context 1, the context conditioning was observed as a highly significant difference of behavioral response amplitudes in two contexts in all groups (panel “after learning”, Figure 3B, *p* < 0.001 for all groups, Wilcoxon Signed Rank Test, *z* = 3.6 for G1, *z* = 3.9 for G2, *z* = 3.5 for G3). On the day following testing of context memory, a session of “reminding” (no shocks, just 20 min in Context 1) and drug injections with or without reminding was performed. Next day testing of long-term context memory demonstrated that ZIP injection without a reminder completely abolished the memory in G1, while ZIP + reminder and scrZIP without reminder had no significant effect on context memory (panel “next day after injections”, Figure 3B, *p* < 0.001 for G2 and G3, Wilcoxon Signed Rank Test, *z* = 3.7 for G2, *z* = 3.4 for G3). The results evidence that blockade of PKMζ results in a loss of consolidated memory, while the same blockade in conditions of reminding and further reconsolidation of memory (which is assumed to take place in several hours) results in maintaining or, more probably, renewal of memory.

We decided to test whether ZIP would be effective at the time after reminding when memory starts to re-consolidate. With the aim to describe the time window of reconsolidation in our model, we repeated the experiments in another groups of snails in conditions that completely repeat conditions of G2 (Figure 3B), but injected ZIP not only 20 min before the reminder, but also at 2 h after the reminder, and at 4 h after the reminder. We have found that injection of ZIP just before the reminding did not influence the snail withdrawal as in the previous series of experiments. At the same time, injection of PKMξ blocker 2 and 4 h after the reminding completely blocked the contextual memory (Figure 3C, G2,G3). Therefore, these results demonstrate with great significance that ZIP injection starts to be effective 2 h and more after the reminder, which completely corresponds to the assumption that long-term memory reconsolidation starts about 2 h after the reminder, and that the molecular substrate for ZIP is formed about this time.

### Electrophysiological Experiments on Isolated CNS

Long-term facilitation (LTF) of excitatory synaptic inputs from sensory neurons to giant premotor interneurons triggering withdrawal in *Helix* is supposed to be a basis of aversion learning and memory in terrestrial snails (Balaban, 2002). We investigated whether the PKMζ takes part in maintenance of LTF in the neural circuit of tentacle and body withdrawal. Normally, the LTF of excitatory synaptic inputs to identified premotor interneurons induced by high-frequency nerve stimulation combined with serotonin bath applications or extracellular stimulation of serotonergic cells lasts at least 4 h (Balaban et al., 2004).

**Figure 4:**
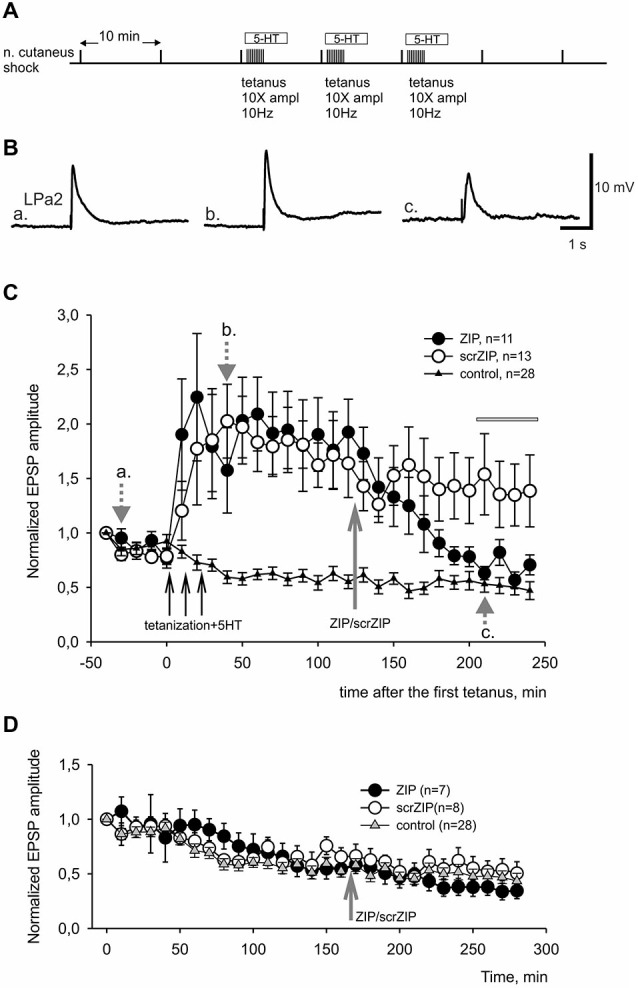
**ZIP reversed facilitation of EPSPs induced by tetanization of homosynaptic input. (A)** Protocol of experiment, tetanization was applied to the same nerve with 10fold increased amplitude. Tetanic stimulation trains were applied along with 10^−5^ M serotonin bath applications. **(B)** Examples of complex EPSPs in response to stimulation of cutaneal nerve at different timing in experiment, marked by letters a, b, c and corresponding gray arrows on **(C). (C)** Averaged results of changes in amplitudes of complex EPSPs after tetanization and after application of ZIP or scrZIP applied at final concentration 2 μM 90 min after the tetanization. In control experiments (triangles) no tetanization and no 5HT was applied, a degree of response decline in several hours experiment was monitored. Averaged data are given as mean ± SEM. **(D)** Averaged results of changes in amplitudes of complex EPSPs after application of ZIP or scrZIP without tetanization and 5HT. Note a significant (to the control levels) decrease of EPSPs amplitudes after ZIP, but not scrZIP, applications. Horizontal gray bar indicates time window in which EPSPs where used for statistical analysis.

In present experiments, we compared effects of ZIP or scrZIP on dynamics of LTF of complex excitatory synaptic inputs to premotor withdrawal interneurons induced by high-frequency tetanization (homosynaptic—of the tested nerve, or heterosynaptic—of another nerve) combined with several serotonin bath applications (five applications for heterosynaptically induced LTF and three applications for homosynaptically induced facilitation, see Section Methods). The protocol described above (Figure 4A) typically induced a strong and long-lasting increase in complex EPSP amplitude (examples on Figure 4B, averaged data on Figure 4C, open and closed circles). Repeated nerve stimulation with single stimuli with 10 min intervals without tetanization and without serotonin application (control experiments) produced some habituation of responses which is typical for this preparation (Figure 4C, triangles). We have found that application of ZIP in final bath concentration of 2 μM at 90th min after the end of tetanization led to a significant decrease of complex EPSPs amplitudes, almost returning them to the level of nontetanized control 80 min after application (Figure 4C, filled circles). At the same time, application of scrZIP did not influence the LTF (Figure 4C, open circles). Average EPSP amplitude, measured in the interval 210–240 min after the first tetanization in experiments with scrZIP application was 140.6 ± 15.8% of baseline before the tetanization, whereas at the same time period, under ZIP application the averaged EPSP amplitude significantly decreased to 68.1 ± 4.5%, (*p* < 0.001, Mann-Whitney rank sum test). However, it was still significantly (*p* < 0.01) greater than averaged amplitude of responses in control (non-tetanized) experiments (46.5 ± 3.2%). In order to check if ZIP/scrZIP can affect the amplitude of non-tetanized EPSPs we performed a separate series of experiments where ZIP/scr ZIP were applied at the same time point as in experiments with LTF but tetanization along with 5-HT treatment were omitted. No significant changes in average EPSP amplitude were found after both ZIP and scrZIP applications in these experiments (Figure 4D). In some experiments, the withdrawal interneurons were impaled with two electrodes and cell input resistance was monitored by applying negative current steps through one electrode while measuring membrane responses with the other one. No significant changes in the cell input resistance were detected after ZIP or scrZIP applications (data not shown).

**Figure 5:**
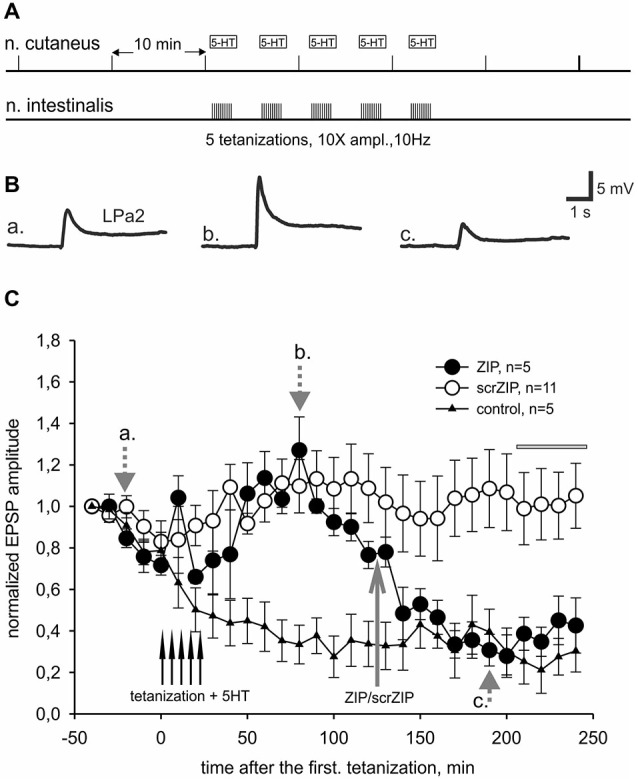
**ZIP reversed facilitation of EPSPs induced by heterosynaptic tetanization. (A)** Scheme of tetanization protocol used for induction of long-term heterosynaptic facilitation of complex EPSPs in giant withdrawal interneurons. Complex EPSPs were elicited by stimulation of cutaneal nerve whereas tetanic stimulation was applied to intestinal nerve along with 10^−5^ M serotonin bath applications. **(B)** Examples of complex EPSPs in response to test stimulation of cutaneous nerve at different timing in ZIP application experiment (filled circles), marked by letters a, b, c and corresponding gray arrows on **(C). (C)** Dynamics of averaged amplitudes of complex EPSPs in experiments with heterosynaptic tetanization + 5HT affected by application of ZIP. Note a significant decrease (to the control levels) of EPSPs amplitudes after ZIP, but not scrZIP (2 μM), applications. In control experiments (triangles) no tetanization and no 5HT was applied, a degree of response decline in several hrs experiment was monitored. Horizontal gray bar indicates time window in which EPSPs where used for statistical analysis.

In our behavioral experiments, the snails were tested by applying the tactile test stimuli to the skin of the head of the animal, which corresponded to cutaneal nerve stimulation in the first series of electrophysiological experiments. However, electrical shocks used in our learning paradigm as negative reinforcement were applied to the rear part of the snail body. Although current was apparently flowing through the whole body of the animal, maximum strength of stimulation definitely was not in the area of the tactile stimulation testing. To reproduce our behavioral situation on the level of isolated CNS more adequately, we performed a series of electrophysiological experiments in which the LTF of EPSPs in parietal giant interneurons, induced by cutaneal nerve stimulation, was elicited by tetanization of the intestinal nerve which innervates caudal part of the snail’s body (heterosynaptic plasticity). In pilot experiments we found that three tetanizations of intestinal nerve combined with 5-HT applications were insufficient to induce stable LTF in Pa2/Pa3 neurons, while five tetanizations induced facilitation lasting for several hours (Figures 5A–C). Similar to results obtained in experiments with homosynaptic facilitation, we have found that application of ZIP 90 min after the last tetanization of intestinal nerve reduced average amplitude of complex EPSPs induced by cutaneal nerve stimulation to the level of non-tetanized controls in the time window 210–240 min after the first tetanization (39.4 ± 4.3% and 26.3 ± 4.7% to initial amplitude, accordingly). Under the scrZIP application, average amplitude of complex EPSPs elicited by test stimuli of cutaneal nerve 210 min after tetanizations was 101.4 ± 8.0% and was significantly larger than the amplitude of responses under ZIP application in the same time period (*p* < 0.01, Figure 5C). Therefore, the results obtained suggest that PKMζ may participate in maintaining heterosynaptic LTF in terrestrial snail nervous system.

### “Artificial Synapse” Experiments

**Figure 6:**
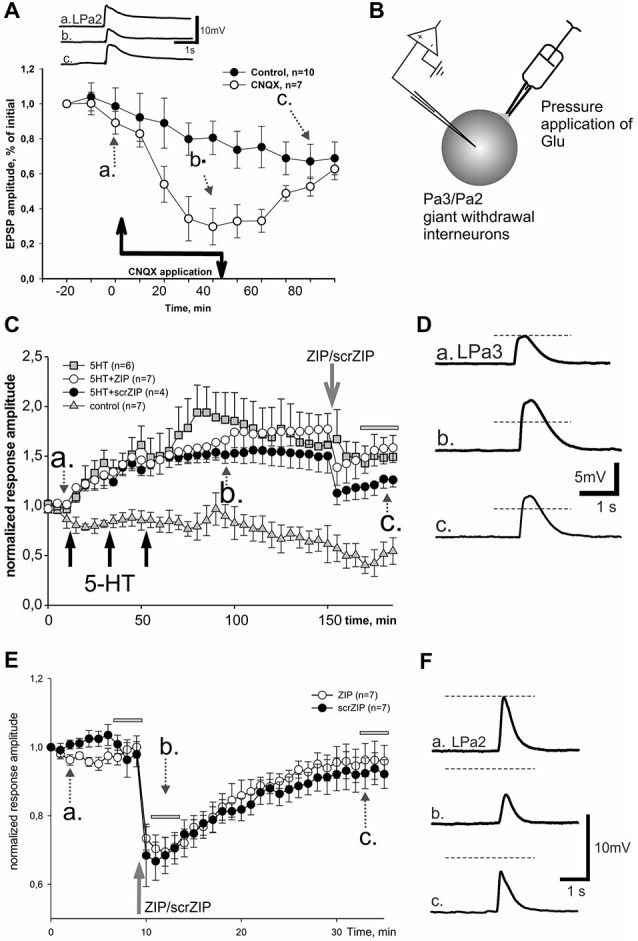
**Effect of ZIP and scrZIP in experiments with Glu responses of the somatic membrane. (A)** In order to prove that glutamate is involved in synaptic responses of giant parietal interneurons, a potent AMPA/kainate antagonist CNQX was applied during repeated cutaneal nerve stimulation, similar to test stimulation in Figures 3, 4. Results evidence involvement of glutamate receptors in these neurons. Control—repeated cutaneal nerve stimulation in experiments without drug applications demonstrating the dynamics of response decline. **(B)** Inset shows the design of experiments in conditions of “artificial synapse”. Rapid removal of Glu from saline was achieved with suction pipette placed near the soma. **(C)** Averaged data from 4 series of experiments demonstrating lack of significant long-term changes of facilitated EPSPs after application of ZIP. Responses to Glu application slowly decreased in control series without any influnces (triangles), while under 3 applications of 5HT for 10 min («pharmacological tetanization») responses slowly and significantly increased in all other experimental series (*p* < 0.001 relative the control at the time window, indicated by the horizontal gray bar, M.-W.). ZIP and scrZIP applications (open circles, closed circles, correspondingly) resulted in small rapid decrease of responses amplitude. Series with 5-HT-induced tetanization only (gray square) served as a control for this condition. Glu was applied 1 per minute, however, each point on the plot represents responses averaged in 5 min intervals (curves are 5x downsampled). **(D)** Inset on the right shows examples of recorded responses, 5HT+scrZIP experiment. **(E)** Additional series of experiments without 5HT application («pharmacological tetanization»), resulting in non-facilitated Glu responses in synaptically isolated parietal withdrawal interneurons. Both ZIP and scrZIP applications induced abrupt and significant short-term drop in response amplitudes in such protocol. Horizontal gray bars indicates time windows in which responses were used for the statistical analysis. No significant difference between ZIP and scrZIP was observed. **(F)** Examples of recordings from ZIP application experiment shown on **(E)**.

As we showed earlier, primary mechanosensory neurons, presynaptic to giant withdrawal interneurons, send their processes to the second cutaneal nerve (Malyshev and Balaban, 2002) and use glutamate as a neurotransmitter (Bravarenko et al., 2003). Therefore it is very likely that complex EPSPs, induced by stimulation of cutaneal nerve in our current experiments, are mediated by glutamate, at least partially. To test this suggestion we performed series of experiments with pharmacological blockade of glutamatergic synaptic transmission. We have found that application of competitive AMPA/kainate receptor antagonist CNQX in a final bath concentration 5 × 10^−6^ M greatly reduced the amplitude of complex EPSPs induced by second cutaneal nerve stimulation (Figure 6A). Washout of CNQX showed slow restoration of responses to control levels. Obtained data (Figure 6A) demonstrate significant involvement of Glu in synaptic responses elicited by stimulation of second cutaneal nerve. Similar results were obtained for the complex EPSPs induced by intestinal nerve stimulation (data not shown).

In order to test the involvement of postsynaptic mechanisms in the maintenance of LTF independently of the presynaptic ones, we designed experiments in which the transmitter was directly applied to somatic membrane of “synaptically isolated" (see Section Methods) giant withdrawal interneurons Pa2 or Pa3 (see inset on Figure 6B). Earlier it was shown in *Helix* that applications of serotonin in such an “artificial synapse” preparation lead to LTF of Glu-induced responses (Balaban et al., 2004). Averaged results demonstrate that in control experiments Glu applications at a 1/min rate elicit slow habituation of responses (Figure 6C, triangles; examples shown in Figure 6D). Three applications of serotonin in final bath concentration 10^−5^ M for 10 min, with a following 10 min washout led to a highly significant increase of Glu-PSPs (Figure 6C, squares). Adding ZIP (in final bath concentration 2 μM) at the 90th min after the end of the last serotonin application resulted in an abrupt and transient decrease of the Glu responses amplitudes followed by a slow increase (Figure 6C, open circles). Finally, average amplitudes of responses, facilitated by serotonin application in the time window 170–185 min from the start of experiment were 147.8 ± 9.3% of baseline and did not differ significantly from the facilitated response amplitudes in the same time window under ZIP application (155.2 ± 7.3%). Surprisingly, we have found that application of scrambled ZIP in the experiments with serotonin-induced facilitation of Glu responses also produced a similar short-term drop in the response amplitudes (Figure 6C, filled circles).

**Figure 7:**
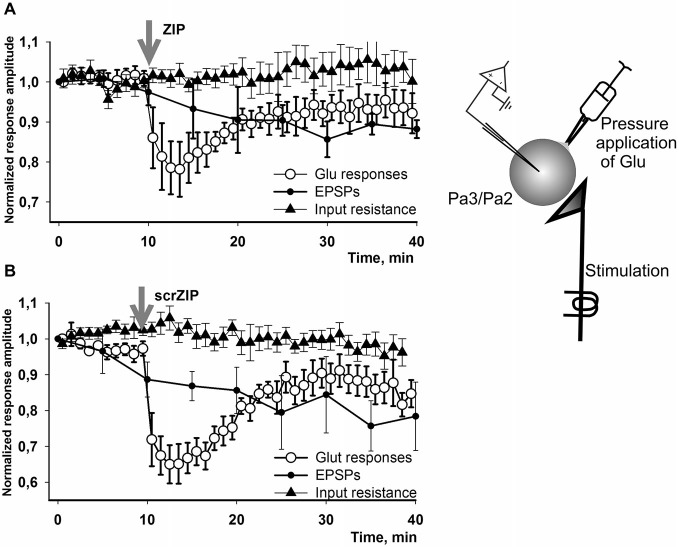
**Effect of ZIP and scrZIP in experiments with Glu responses of the somatic membrane in conditions of preserved synaptic inputs**. In a special series of experiments on isolated CNS in configuration which was used in the main series of our experiments with homo- and heterosynaptic facilitation (Figures 3, 4) we applied test pulses to the second cutaneal nerve eliciting EPSPs in parietal interneurons every 5 min. Between regular nerve stimulations we applied pulses of Glu to the soma of the same neuron every minute. Before each test stimulus the input resistance of the recorded cell was measured. **(A,B)**—applications of ZIP or scrambled ZIP in concentrations 2 μM produced a transient drop in Glu-responses in withdrawal interneurons that do not affect the EPSPs elicited by the cutaneous nerve stimulation in the same cell. No significant changes were observed in the input resistance throughout the experiment. EPSPs showed some habituation which is typical for this preparation. Note that the amplitudes of Glu responses in 20 min were restored almost to the level existing before ZIP/scrZIP application.

We have performed additional series of experiments with non-facilitated Glu responses recorded in somata of parietal interneurons and found that both ZIP and scrZIP induced abrupt and significant (but short-term) drops in response amplitudes from 98.8 ± 1.6–70.2 ± 2.1 for ZIP and from 98.4 ± 2.2–68.6 ± 2.9 for scrambled ZIP (Figures 6E,F). In 20 min after ZIP or scrZIP applications, amplitudes of the responses were restored to 96.1 ± 2.7% and 92.8 ± 2.5%, accordingly. As it was described in the Methods section, experiments with Glu-induced responses were carried out in “synaptically isolated” preparations, consisting of upper segments of parietal ganglia containing bodies of parietal withdrawal interneurons. It is possible that the transient inhibiting effect of ZIP/scrZIP application was an artifact of this type of preparation. In order to clarify this situation we carried out a special series of experiments on isolated CNS in the configuration which was used in the main series of our experiments with homo- and heterosynaptic facilitation but added repeated measurements of input resistance of the recorded cell and Glu application on the soma (see inset on Figure 7). We applied test pulses to the second cutaneous nerve eliciting complex EPSPs in withdrawal interneurons every 5 min. Between regular nerve stimulations we applied pulses of Glu to the soma of the same neuron every minute. We have found that applications of ZIP or scrambled ZIP in concentrations of 2 μM produced a transient drop in Glu-responses in parietal interneurons but no changes in the amplitude of complex EPSPs elicited in the same cell by the cutaneal nerve stimulation or any changes in input resistance (Figures 7A,B). Therefore, it appears that both peptides (ZIP and scrambled ZIP) have some transient non-specific effects on the responses induced by the application of glutamate on the somatic membrane of parietal withdrawal interneurons. Extrasynaptic glutamate receptors are presumably involved in these transient changes.

## Discussion

### Activity of PKMζ Homolog Maintains Associative Long-term Memory in *Helix*

In our experiments it was shown that a homolog of PKMζ exists in the neural transcriptome, and that the molecules immunoreactive to the conservative sites of PKMζ are present in the neurons and neuropile of *Helix lucorum*. In behavioral experiments it was shown that associative contextual fear memory disappeared under blockade of PKMζ by a selective inhibitor ZIP, but not under scrambled ZIP. If ZIP application was combined with a “reminder” (20 min in “noxious” context, no shocks), no impairment of the long-term context memory was observed. Testing of ZIP effectiveness at the time after reminding when memory starts to re-consolidate demonstrate that ZIP injection starts to be effective 2 h and more after the reminding, which completely corresponds to the assumption that long-term memory reconsolidation starts about 2 h after the reminder and it is the time when the molecular substrate for ZIP is formed (Debiec et al., 2002; Duvarci et al., 2005). Obtained results suggest that blockade of PKMζ impairs existing fear memory in terrestrial snails, while the “reminder” addresses this memory and starts the process of its reconsolidation even under transient (estimated time of ZIP presence in active state in the hemeolymph is not more than 4 h) blockade of PKMζ synthesis. It was recently shown in *Helix* that 4 h after the reminder under protein synthesis blockade, the memory still exists, but is completely absent in the same animals 24 h later (Balaban et al., 2014). A recent study in marine mollusk *Aplysia* can help in explaining such a profile of protein-dependency of memory maintained by atypical PKM (Bougie et al., 2012). To activate cleavage of PKM Apl III isoform, activity-induced protein synthesis of calpain is required at the early phase of memory formation.

### Activity of PKMζ Homolog Maintains Long-term Facilitation in *Helix*

In electrophysiological experiments we tried to prove that PKMζ takes part in maintenance of LTF in the neural circuit underlying tentacle withdrawal. LTF of excitatory synaptic inputs to giant parietal premotor interneurons was induced by high-frequency stimulation combined with serotonin bath applications and lasted at least 4 h. We found that bath application of 2 × 10^−6^ M ZIP at 90th min after the tetanization reduced the EPSP amplitude to the non-tetanized EPSP values. Application of scrambled ZIP peptide at similar time and concentrations didn’t affect the EPSP amplitudes. Our results largely correspond to published in *Aplysia* (Cai et al., 2011) effects of ZIP on LTF of synaptic connections between co-cultured sensory and motor neurons as well as to data obtained in rodents where ZIP disrupted persistence of LTP in hippocampus (Ling et al., 2002; Pastalkova et al., 2006). Therefore, obtained here results support the idea of atypical PKC involvement in post-induction maintenance of long-term plasticity in *Helix*.

### PKMζ Homolog is not Involved in LTF of “Artificial Synapse” in *Helix*

In order to test whether effects of ZIP can be reproduced in a model situation, we performed experiments with LTF of somatic membrane responses to local glutamate applications. It was shown earlier that serotonin applications in such “artificial synapse” conditions elicit LTF of responses (Balaban et al., 2004). In the present study it was shown that ZIP had no effect on LTF in these conditions, which may be explained by the very low concentration of PKMζ molecules in somata of these identified neurons, as was evidenced by immunochemistry. Transient (minutes) decrease of the response to glutamate under ZIP and scrZIP is described for the first time and it may be of interest to investigate the mechanism of this phenomenon at the channel level.

The drugs were introduced via intrahemeocoel injections and had access to the entire CNS. Therefore, although facilitation of synaptic inputs to the withdrawal interneurons in the parietal ganglia has been demonstrated to mediate long-term sensitization (Balaban, 2002), and although we have shown here that inhibition of PKMζ disrupts maintenance of both long-term contextual aversive memory *in vivo* and LTF of the synaptic inputs to the parietal withdrawal interneurons *in vitro*, we cannot rule out the possibility that our behavioral results were attributable, at least in part, to actions of ZIP on central sites other than the synapses between sensory neurons and interneurons. The present results add to the accumulating evidence that PKMζ plays crucial roles in the persistence of long-term memory and long-term synaptic plasticity in both vertebrates and invertebrates (Drier et al., 2002; Ling et al., 2002; Serrano et al., 2005; Pastalkova et al., 2006; Shema et al., 2007).

### Possible Mechanism of PKMζ-Mediated Maintenance of Synaptic Facilitation in *Helix*

How does PKMζ maintain synaptic facilitation which we believe underlie associated long-term memory in *Helix*? According to T. Sactor’s model, the increase in synaptic strength in mammalian models of synaptic plasticity is based on PKMζ-mediated increase in trafficking of AMPA receptors into the postsynaptic membrane (Sacktor, 2012). As it was shown in Aplysia, mostly by data from D. Glanzman’s laboratory, up-regulation of trafficking of AMPA receptors plays crucial role in the maintenance of both synaptic plasticity and behavioral learning in this mollusk (Chitwood et al., 2001; Li et al., 2005; Glanzman, 2010). Induction of associative learning as well as induction of synaptic facilitation in our experiments, similar to the situation in *Aplysia*, relies heavily on serotonin modulation of glutamatergic synapses. By application of selective blockers it was demonstrated that synaptic inputs to withdrawal interneurons, which are subject to change during induction of avoidance learning in terrestrial snails (Balaban, 2002), are mediated by glutamate (Bravarenko et al., 2003). Therefore, it is very likely that synaptic facilitation in our experiments is also mediated by constant increase of insertion of AMPA-like receptors into the postsynaptic membrane. We can further hypothesize that serotonin, which was used for induction of long-term synaptic facilitation in *Helix*, induced calpain-mediated cleavage of *Helix* ortholog of PKCζ to form PKMζ which underlies increased trafficking of AMPA receptors. Such a mechanism was recently reported for *Aplysia* (Bougie et al., 2009, 2012) and we believe it can also play a role in the mechanisms of synaptic plasticity in *Helix*. It should be noted here that *Aplysia* PKMζ (and presumably in *Helix* as well) is not formed by alternative translation from mRNA of an atypical isoform of PKC as it happens in mammals (Hernandez et al., 2003), but rather, it is produced by calpain-dependent cleavage of PKCζ ortholog (Bougie et al., 2009).

### Specificity of ZIP and the Role of PKMζ in Learning and Memory

Nowadays, much evidence suggests an involvement of PKMζ in different types of synaptic plasticity and learning and memory in rodents, insects and mollusks (for review see, for example, Sacktor, 2012). Recently, in the cockroach it was shown that systemic injections of either chelerythrine or ZIP erase long-term olfactory memories, but have no effect on memory acquisition during conditioning (Deng et al., 2015). On the other hand, several other groups reported that activity of PKMζ might not be necessary for synaptic plasticity. Thus, it was demonstrated that mice with a knockout for the PKMζ gene exhibit normal hippocampal synaptic plasticity, learning and memory (Lee et al., 2013; Volk et al., 2013), suggesting existence of other targets for ZIP, presumably the isoforms of PKMζ. It was also reported that scrambled ZIP with a changed amino-acid sequence can also block PKMζ, although not as effectively as ZIP itself (Lee et al., 2013). In our experiments, scrZIP was not able to reverse potentiated EPSPs to the base level, and was also unable to erase memory in the learned snails. However, we observed similar transient reduction of glutamate responses, evoked by application of glutamate to the soma of neurons, both by ZIP and scrZIP, which suggests existence of some similar properties in these two peptides. Nevertheless, despite disagreement on the role of PKMζ itself in processes of learning in memory, nobody argues that the short peptide ZIP can erase stored memories in a variety of experimental animals—from mollusks to mammals. This fact, without any doubts, requires future investigations.

### PKMζ Expression in CNS

Using the sc-216 antibody, PKMζ was detected previously in membranes, cytoplasm and in the nucleus of cultured non-nervous PC12 cells (Crisanti et al., 2005). In embryonic hippocampal neurons, both sc-216 and sc-17781 antibodies detected PKMζ both in cell bodies and at non-axon-forming neurites (Parker et al., 2013). This pattern of staining coincides with our data. In adult rat brain, it was shown that PKMζ is widely expressed in the forebrain including hippocampus and neocortex (Hernández et al., 2013). It was detected in the cell somata and dendrites, including postsynaptic densities. We suggest that these results match to the granules we have seen in cultured neurons of the snail. Thus, cell soma staining (granules) and absence of staining in axons seems to be a common case within different brains. Therefore, our data show similar patterns of PKMζ immunoreactivity distribution in the neurons of rodents and mollusk which suggests a common role for PKMζ in neural functions in these two classes of animals.

## Conclusion

We have demonstrated that inhibiting an isoform of PKMζ in terrestrial snail eliminates the long-term memory, as well as the specific form of long-term synaptic plasticity that underlies the learning in the well-studied invertebrate *Helix*.

## Conflict of Interest Statement

The authors declare that the research was conducted in the absence of any commercial or financial relationships that could be construed as a potential conflict of interest.
